# Dynamics of Biofilm Regrowth in Drinking Water Distribution Systems

**DOI:** 10.1128/AEM.00109-16

**Published:** 2016-06-30

**Authors:** I. Douterelo, S. Husband, V. Loza, J. Boxall

**Affiliations:** Pennine Water Group, Department of Civil and Structural Engineering, The University of Sheffield, Sheffield, United Kingdom; University of Minnesota

## Abstract

The majority of biomass within water distribution systems is in the form of attached biofilm. This is known to be central to drinking water quality degradation following treatment, yet little understanding of the dynamics of these highly heterogeneous communities exists. This paper presents original information on such dynamics, with findings demonstrating patterns of material accumulation, seasonality, and influential factors. Rigorous flushing operations repeated over a 1-year period on an operational chlorinated system in the United Kingdom are presented here. Intensive monitoring and sampling were undertaken, including time-series turbidity and detailed microbial analysis using 16S rRNA Illumina MiSeq sequencing. The results show that bacterial dynamics were influenced by differences in the supplied water and by the material remaining attached to the pipe wall following flushing. Turbidity, metals, and phosphate were the main factors correlated with the distribution of bacteria in the samples. Coupled with the lack of inhibition of biofilm development due to residual chlorine, this suggests that limiting inorganic nutrients, rather than organic carbon, might be a viable component in treatment strategies to manage biofilms. The research also showed that repeat flushing exerted beneficial selective pressure, giving another reason for flushing being a viable advantageous biofilm management option. This work advances our understanding of microbiological processes in drinking water distribution systems and helps inform strategies to optimize asset performance.

**IMPORTANCE** This research provides novel information regarding the dynamics of biofilm formation in real drinking water distribution systems made of different materials. This new knowledge on microbiological process in water supply systems can be used to optimize the performance of the distribution network and to guarantee safe and good-quality drinking water to consumers.

## INTRODUCTION

Drinking water distribution systems (DWDS) support a diverse microbial community attached to the pipe walls where biofilms form. It is accepted that if the hydraulic conditions in the distribution network change and overcome biofilm adhesive forces, biofilms can detach from the pipe walls, and they have the potential to impact the performance of the water infrastructure and the final quality and safety of the supplied water (1, 2). Different parameters within DWDS might influence the way the material and biofilms accumulate on the pipe walls. For example, pipe characteristics (e.g., material, roughness, etc.) might affect biofilm composition and morphology, and hydrodynamic conditions can affect the strength of attachment to pipes and physicochemical characteristics of the source water and the type of microorganisms inhabiting biofilms (3–8). However, limited knowledge exists on the biofilm dynamics in real DWDS, since these systems are difficult and costly to access, restricting most of the studies of microbial diversity in these ecosystems to samples from taps and water meters (9, 10) and/or to biofilms developed for a limited time in artificial systems (11, 12). Considering that biofilms degrade water quality and safety through the potential hosting of undesirable microorganisms (13, 14), realistic and *in situ* research on biofilm formation in DWDS is essential to understand the factors that influence their development. Similarly, further research is needed to assess the impact of biofilm mobilization during the supply of drinking water, as this is known to contribute to discoloration, which is the single largest cause of customer contacts relating to water quality.

Biofilm growth in DWDS is a concern for water companies, which spend significant effort and resources on monitoring and control strategies to minimize the risk that biofilms pose to the delivery of high-quality safe drinking water (13). However, biofilms are difficult and practically impossible to eliminate from DWDS surfaces due to the protection offered by the microbial self-produced extracellular polymeric substance (EPS) matrix. This matrix protects microorganisms within biofilms from external adverse factors and fluctuations, including chemical disinfection (15).

Cleaning techniques, such as flushing, are commonly used by water companies to remove material attached to pipes and to prevent discoloration issues (16, 17). Flushing of the pipes raises the system shear stress, causing mobilization of particulate material from the pipe walls into the bulk flow. Despite the short-term prevention of water quality issues, this method is not capable of eliminating all the biofilm attached to the pipes, and bacteria can grow from the remaining material (7). Consequently, knowledge of the microbiological composition of material mobilized from pipes and the potential for biofilm regrowth is necessary to improve control and management strategies in DWDS.

The aim of the research reported here is to ascertain material accumulation and mobilization patterns in DWDS and to better understand the performance risks and impacts of these assets on the quality of the supplied drinking water.

The specific objectives of this research were to (i) establish if flushing can act as a structuring force to shape bacterial communities, (ii) determine how bacteria were populating the distribution network after repeated periodical flushing, and (iii) determine if the accumulation of material attached to pipe walls is affected by seasonal changes in bulk water and/or by pipe material.

## MATERIALS AND METHODS

In order to understand the accumulation potential and the dynamics of microbial communities attached to the inner surfaces of pipes within a DWDS, seasonal flushing and associated sample analysis were undertaken. Repeated flushing events were imposed within a district metered area (DMA), a defined distribution zone isolated by closed valves for which the quantities of water entering and leaving are measured. Water samples were collected before and during flushing operations to provide samples representative of material from the background flux as well as material mobilized from the pipe walls.

### Field site and sampling.

**FIG 1:**
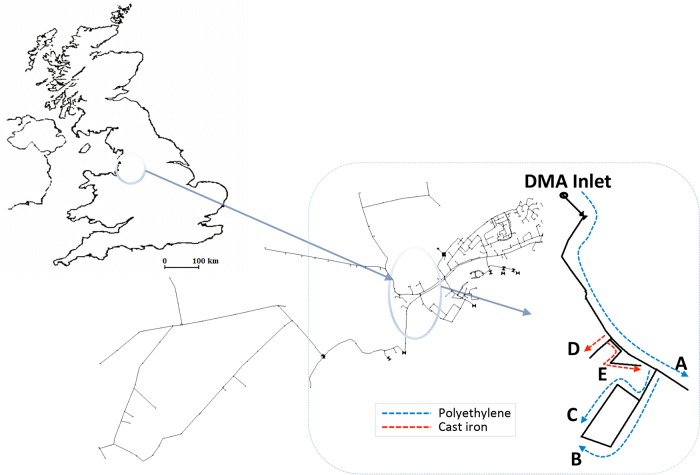
Schematic showing the location of the DMA in Northwest England and the layout and characteristics of the flushing sites. (United Kingdom map adapted from reference 53, with permission from Elsevier.)

**FIG 2:**
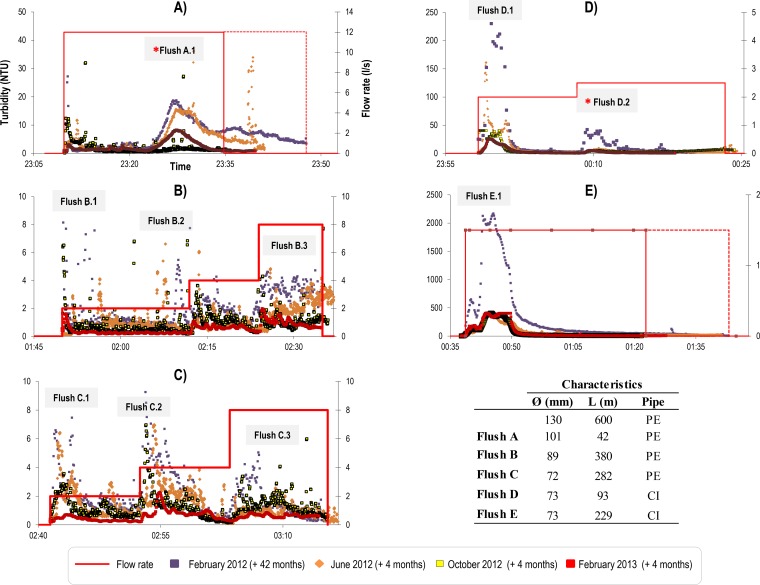
Turbidity and flow rate time series for the repeated flushing operations. Hydrant discharge flow rate (in liters per second) is indicated by a line, and the measured turbidity response is coded according to date of operation. Letters indicate the different flushes, and numbers indicate the flushing steps. Samples from all the flushing interventions were used for fingerprinting analysis (T-RFLPs), but those used for Illumina sequencing are indicated with an asterisk.

The trial site was part of a large DMA in the Northwest of England (Fig. 1) supplied by abstracted river water with aluminum sulfate coagulation treatment and fed from a 1.8-m-diameter lined trunk main. The first trial was conducted 42 months after the last recorded disturbance in this DMA (a previous monitored flushing trial using the same protocol). Subsequent flushes were carried out at 4-month intervals to investigate seasonal source water effects. The flushing duration, flow rates, and characteristics of the pipes are shown in Fig. 2. In total, 5 sections were flushed, with 3 plastic pipes (Fig. 2A to C) and 2 cast iron pipes (Fig. 2D and E).

Flushing was undertaken in a sequential unidirectional manner using strategic valve closures that maintained a clean water front and ensured that flushing flows exceeded typical daily peak values. Control was achieved by monitoring the flow rate at the fire hydrant used for flushing. The trials were conducted at night to minimize both potential customer impacts and demand fluctuations. Noncontrollable demand fluctuations would compromise the ideally constant and repeatable flushing-induced target shear stress. The maximum possible flushing flow rate was defined by hydraulic analysis of the upstream trunk main to ensure that no upstream mobilization effects were anticipated. This analysis showed typical daily peak flows exceeding 20 liters/s, with nighttime flow rates of 2 to 4 liters/s. Thus, the first polyethylene pipe section was limited to the highest flushing flow rate of 12 liters/s. Turbidity monitoring at the DMA inlet during operations confirmed that the flushing operation impact was limited to the trial sections. Subsequent sections were flushed at lower maximum rates to prevent any additional upstream material mobilization. Flushing was planned to be in increments above the normal daily peak in flow for each pipe length, to both control and investigate the mobilization of material due to increasing imposed force. Due to the high daily flow, the limiting maximum value from the upstream trunk main, and the volume of water to be discharged (pipe volume, 9.4 m^3^), only a single flushing flow rate was possible in pipe length A. Stepped flushing was achieved for pipe lengths B and C, both of which were plastic pipe materials. Flushes D and E, affecting cast iron pipe lengths, were similarly planned; however, while on site, it was discovered that the obtainable flows were significantly lower than indicated by hydraulic modeling. Hence, only a small second increment was achieved during flush D and a single rate for flush E. These lower-than-anticipated flows were subsequently attributed to poor internal pipe conditions due to ongoing corrosion processes (18).

Water samples with total volume of 5 liters were collected before the flushing (preflush water samples) and then after each incremental increase in flow at one turnover of the pipe volume to collect the anticipated mobilized peak material. Samples from the inlet, all 5 preflushes, and 10 during-flush steps were used for bacterial community fingerprinting by terminal restriction fragment length polymorphism (T-RFLP) analysis (Fig. 2A to D). For the detailed bacterial community analysis using Illumina sequencing, two sections were selected from the trials, flush A.1 (Fig. 2A) and flush D.2 (Fig. 2D).

### Water analysis: online turbidity monitoring, physicochemical analysis, and heterotrophic plate counts.

Turbidity (in nephelometric turbidity units [NTU]) was measured using ATI A15/76 turbidity monitors (Analytical Technology, Inc., United Kingdom) connected to the flushing standpipe and hydrant sample taps at strategic points in the DMA (i.e., at the DMA inlet to confirm no mobilization of material from upstream). Several parameters were measured *in situ* during the trials, matching discrete water sample collection times. pH and temperature were determined using a Hanna HI 991003 portable meter and probe, and free chlorine was determined using an HI 96711 photometer (Hanna Instruments, Leighton Buzzard, United Kingdom). Other parameters were determined by United Utilities Scientific Services, in a United Kingdom-accredited drinking water laboratory, and in accordance with drinking water regulation requirements, as previously described by Douterelo et al. (8). Bacterial colony counts were determined by the pour plate method by United Utilities Scientific Services, in accordance with the United Kingdom Environment Agency recommendations (20). Cultures were incubated at 37°C for 48 h (2-day colony) and 22°C for 72 h (3-day colony), and colonies were counted after that period of time.

### Molecular analysis of microbial communities. (i) DNA extractions.

A total of 60 bulk water samples of 5 liters, including inlet, preflushing, and during-flushing samples, were collected. From these samples, three replicates of 1 liter were filtered via a sterile filtration unit using 0.22-μm-pore-size nitrocellulose membrane filters (Millipore Corp.). DNA extraction from the filters was carried out by a method combining proteinase K digestion and a standard phenol-chloroform-isoamyl alcohol extraction (21). The quantity and purity of the extracted DNA were assessed using a NanoDrop ND-1000 spectrophotometer (NanoDrop, Wilmington, DE, USA). Those samples with high levels of DNA contamination (mainly metals) were cleaned using a Wizard DNA clean-up system (Promega, United Kingdom).

### (ii) T-RFLPs.

A fragment of approximately 490 bp of the bacterial 16S rRNA gene, targeting regions V1 to V3, was amplified using primers 63F (5′-CAGGCCTAACACATGCAAGTC-3′) and 518R (5′-CGTATTACCGCGGCTGCTCG-3′) (22). The 63F primer was labeled at the 5′ end with the phosphoramidite dye 6-carboxyfluorescein (FAM) (Applied Biosystems, United Kingdom). The PCR mixture and conditions were as previously described by Douterelo et al. (19). Not all DNA samples (*n* = 180) yielded good PCR amplification (low quantity or quality), and the inadequate samples were excluded from further analysis. In total, 132 amplicons representing each of the seasonal flushing interventions were analyzed to obtain a fingerprint of the microbial communities recovered by flushing the DMA. Amplicons were purified using the QIAquick PCR purification kit (Qiagen, Inc., Valencia, CA) and then digested at 37°C for 2 h with the restriction enzyme AluI (Roche Diagnostics, Burgess Hill, United Kingdom). Desalted restriction digests were mixed with 1 μl of deionized formamide and 0.5 μl of a ROX-labeled GeneScan 500-bp internal size standard (Applied Biosystems), denatured at 94°C for 3 min, and immediately transferred to ice. T-RFLPs were separated by capillary electrophoresis using an automated ABI3730 genetic analyzer (Applied Biosystems). Differences in the length and abundance of T-RFLPs were resolved by a comparison with the known-size internal standard, and the fragment sizes were estimated by interpolation using a Local Southern algorithm with the software GeneMapper 3.7 (Applied Biosystems). Fragments <50 bp and with a peak height of <50 were considered background noise and eliminated from the analysis.

To analyze the obtained profiles, T-RFLPs were aligned on the basis of fragment lengths and peak areas using the T-Align software (23). From the aligned T-RFLPs, a Bray-Curtis similarity matrix was calculated and then visualized by multidimensional scaling (MDS) diagrams using Primer version 6 (Primer-E, Plymouth, United Kingdom). The similarity matrix was used to test the significance of differences among samples based on pipe material and seasonality by analysis of similarity statistics (ANOSIM).

### (iii) Illumina MiSeq sequencing.

Selected samples (*n* = 24) from one plastic (Fig. 2A, flush A.1) and one cast iron pipe section (Fig. 2D, flush D.2) were used for detailed analysis of the dynamics and differences in bacterial communities between pipe materials. These two pipe lengths were selected based on the T-RFLP data and to minimize the influence of any effects from other upstream pipe lengths within the DMA. These were the initial pipe lengths into the DMA, with cast iron pipe length D being a branch from plastic pipe length A. Sequencing was performed by use of the Illumina MiSeq technology with the paired-end protocol by Research and Testing Laboratories (Lubbock, TX, USA) using primers 28F (GAGTTTGATCNTGGCTCAG) and 519 (RGTNTTACNGCGGCKGCTG). Paired-end reads were merged and denoised via the Research and Testing Laboratory Pipeline to remove short sequences, singletons, and noisy reads. Chimeras were detected using UCHIME (24) and removed from further analysis. The sequences were clustered in operational taxonomic units (OTU) and selected using UPARSE (25). Taxonomic assignments were made with the USEARCH global alignment program (26).

The OTU table in biom format was then used in Quantitative Insights into Microbial Ecology (QIIME) (27) to calculate rarefaction curves. Alpha diversity was estimated using a rarefaction analysis (number of OTU observed versus number of sequences sampled) performed at a 97% sequence similarity cutoff for each sample. Calculated collector's curves for the Chao1 richness estimator (28) and Shannon diversity index (29) were obtained for all the sequences.

The data on relative bacterial abundance at the species level were transformed by square root calculations, and a Bray-Curtis similarity matrix was generated. Subsequently, complete-linkage clustering, a method of agglomerative hierarchical clustering, was used to group the samples according to relative bacterial abundance. To evaluate the statistical significance of the cluster generated from the analysis, a similarity profile analysis (SIMPROF, 999 simulations) was carried out using the software Primer version 6 (Primer-E, Plymouth, United Kingdom).

### Statistical analysis of physicochemical factors and microbiology.

Nonparametric Spearman's rank correlation analysis was carried out using the software IBM SPSS Statistics 21.0 to establish relations between physicochemical parameters in the water, bacteriological indicators (richness and diversity), and the relative abundances of the most representative bacterial phyla in the samples. The results are shown in Fig. S1 in the supplemental material.

### Accession number(s).

Sequencing data were deposited in the National Center for Biotechnology Information (NCBI) Sequence Read Archive (SRA) under accession no. SRP072801.

## RESULTS

### Turbidity and flushing events.

Turbidity values were highest for the first flush following 42 months of normal operation (no recorded disturbances) in both plastic and cast iron pipe sections (Fig. 2). In one cast iron pipe length, turbidity reached an exceptionally high value of >2,000 NTU (Fig. 2, flush E.1), while the highest recorded value in the plastic pipes for this initial trial was around 20 NTU. However, it should be noted that the instrument response was calibrated to only 400 NTU, and turbidity measurements may be nonlinear above this. As all pipe lengths are interconnected and near the single-source DMA entry, this suggests that corrosion of the cast iron pipe is a dominant source of material, as reported by Husband and Boxall (17). This is supported by the high values of iron measured from discrete sample collection (Table 1). The subsequent flushing trials in the cast iron pipe lengths following each 4-month accumulation period produced very similar yet significant turbidity responses (Fig. 2D and E). Despite similar temporal patterns of turbidity, samples from the plastic pipe lengths showed higher turbidity levels in February 2012 (anticipated due to longer accumulation period) and June 2012, and lower levels were observed in October 2012 and February 2013 (Fig. 2A to C).

**TABLE 1:** Results of physicochemical analysis of bulk water samples showing the characteristics of the water from the pipe sections^*a*^

Sample by type and date (mo/yr)	Temp (°C)	pH	Free Cl_2_ (mg/liter)	Aluminum (μg/liter)	Iron (μg/liter)	Manganese (μg/liter)	Nitrite as N (mg/liter)	Nitrate as N (mg/liter)	TOC (mg/liter)	PO_4_ total (μg/liter)	Sulfate as SO_4_ (mg/liter)	2D colonies at 37°C (no./ml)^*b*^	3D colonies at 22°C (no./ml)^*c*^
Inlet													
Feb-12	6.4	7.17	0.45	10.9	5.2	0.20	0.0029^*d*^	2.95	1.66	1,460	45.7	0	0
Jun-12	18.0	6.40	0.46	3.4	7.1	0.32	0.0006^*d*^	1.24	1.43	1,020	42.3	0	0
Oct-12	10.0	6.97	0.64	16.0	115.0	0.90	0.0006	1.66	1.47	1,260	43.6	0	1
Feb-13	6.0	8.45	0.82	10.6	34.5	1.70	0.0006^*d*^	2.69	1.36	1,600	56.5	0	0
Plastic pipe													
Preflush A													
Feb-12	6.1	7.07	0.33	340.0	1,380	12.70	0.0029^*d*^	3.06	1.87	1,760	47.2	10	17
Jun-12	17.5	6.74	1.03	6.0	43.8	2.90	0.0006^*d*^	1.28	1.37	1,500	43.3	0	2
Oct-12	8.5	7.25	0.85	12.1	6.6	0.90	0.0006	1.84	1.63	1,570	43.8	0	0
Feb-13	4.8	8.76	0.76	31.6	63.6	6.65	0.0006^*d*^	2.55	1.35	1,490	55.4	0	1
Flush A.1													
Feb-12	6.3	7.12	0.44	1,300	2,020	44.80	0.0029^*d*^	2.90	1.71	2,690	45.9	0	0
Jun-12	16.3	7.00	1.20	321	88	13.30	0.0006^*d*^	1.23	1.33	1,690	42.3	0	0
Oct-12	8.4	7.36	0.82	71	14	3.70	0.0006	1.69	1.47	1,500	43.0	0	0
Feb-13	4.1	8.02	0.64	58.6	42.7	7.47	0.0006^*d*^	2.71	1.29	1,410	56.3	0	52
Cast iron pipe													
Preflush D													
Feb-12	6.0	7.10	0.42	3,120	3,570	135.00	0.0029^*d*^	2.88	1.84	5,210	46.0	0	0
Jun-12	16.5	7.07	0.07	226	689	11.60	0.0008	1.24	1.39	1,640	42.6	0	6
Oct-12	7.9	7.42	0.71	14.6	47	0.90	0.0006	1.57	1.53	1,420	44.3	0	0
Feb-13	4.5	8.90	0.79	3.4	34.7	0.57	0.0006^*d*^	2.66	1.47	1,530	55.8	0	0
Flush D.1													
Feb-12	6.4	7.09	0.43	1,420	2,300	116.00	0.0029^*d*^	2.91	1.73	3,460	45.5	0	0
Jun-12	16.4	7.08	0.09	186	154	13.40	0.0006	1.25	1.51	1,420	42.4	0	0
Oct-12	8.5	7.51	0.72	84.1	166	5.70	0.0006	0.00	1.55	2,320	43.1	0	0
Feb-13	4.3	8.60	0.70	93.2	476	19.10	0.0006^*d*^	2.61	1.32	2,130	55.6	0	1
Flush D.2													
Feb-12	6.2	7.07	0.40	892	2,360	166.00	0.0029^*d*^	2.94	1.68	3,040	45.3	0	1
Jun-12	15.9	7.07	0.26	332	423	56.30	0.0006	1.27	1.38	1,840	42.1	0	0
Oct-12	8.0	7.54	0.78	59.6	733	10.1	0.0006	1.55	1.50	2,290	43.4	0	1
Feb-13	4.3	8.40	0.74	13.3	59	4.13	0.0006^*d*^	2.61	1.28	1,860	55.3	0	3

a Samples used for Illumina sequencing were A.1 and D.2.

b 2D, 2-day.

c 3D, 3-day.

d Below detection limit.

### Water physicochemical analysis and heterotrophic plate counts.

Table 1 shows the data from the physicochemical analysis of the water and the heterotrophic plate counts from pipe sections. Several physicochemical variables showed similar average values during the monitored period at the two sections of pipe and different flushing steps. For example, total organic carbon (TOC) levels ranged between 1.28 and 1.87 mg/liter during all the seasonal flushing interventions. Over the studied period, the pH was near neutral, except for the last flushing campaign (February 2013). Temperature showed seasonal fluctuations, from an average temperature in winter (February 2012 and 2013) of 4.7 to 6.2°C to 8.6°C in October and a maximum temperature in June of 16.8°C. Other parameters also varied over time, such as nitrate levels, which were higher in February than in June. On average, phosphate levels were higher in the cast iron pipes than in plastic pipes and also in samples from the first flush. Higher levels of sulfate were detected in samples from February 2013.

Analysis was conducted for all regulated United Kingdom bacterial parameters with zero detection, providing confidence in the supplied water quality and safety. Colony counts were higher from the plastic pipe in February 2012 (*n* = 10 and 17) and in February 2013 (*n* = 52) than from the cast iron pipe, in which only 6 colonies were detected in June 2012 and 3 in February 2013.

### Community analysis by T-RFLPs.

To determine differences in the bacterial community structures of the material attached to the pipes, terminal restriction fragments were obtained from 132 samples acquired at all the flushing steps during the 4 flushing events taking place from February 2012 to February 2013. Inlet samples were also analyzed, but good amplifications failed for the samples in the last two flushing campaigns (October 2012 and February 2013), and it was not possible to obtain restriction fragments from those. This lack of amplification, or weak amplifications, can be related to slightly higher concentration of iron (Table 1) and potential low bacterial concentration in the inlet samples from the last two sampling campaigns. The DNA cleaning system used to purify contaminated DNA left these samples with insufficient DNA to obtain good amplicons and subsequent restriction fragments.

**FIG 3:**
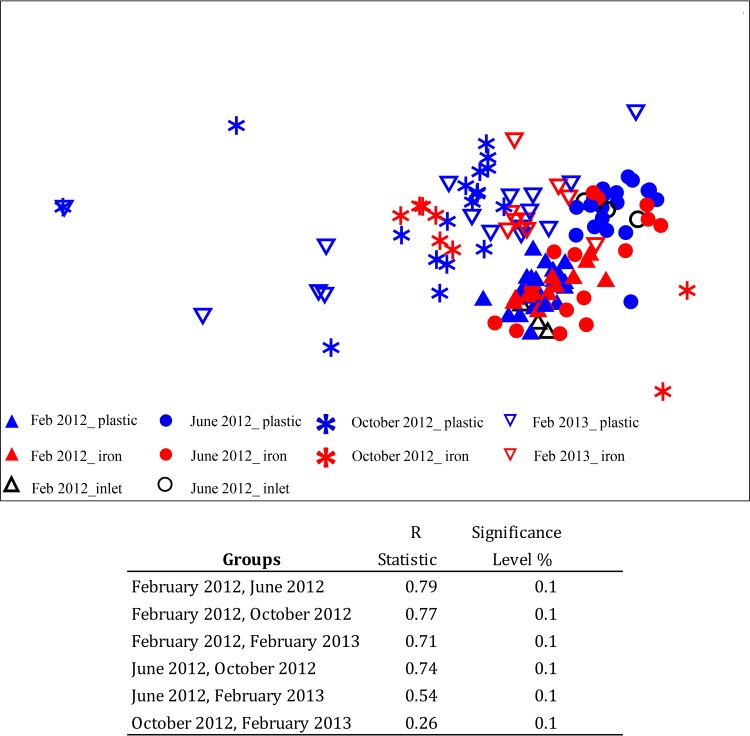
Nonmetric multidimensional scaling (MDS) ordination based on a Bray-Curtis resemblance matrix calculated from T-RFLP profiles. The plot shows the distribution of samples over time and according to pipe material.

Figure 3 shows the results from the nonmetric MDS and the ANOSIM for all the T-RFLP profiles. When all the samples were analyzed together, significant differences (significant level, 0.1%) in the bacterial community fingerprints over time were detected. However, no significant differences between the plastic and cast iron pipe samples were obtained. When samples belonging to a specific flushing event were analyzed, samples tended to distribute according to pipe material, yet these differences were not statistically significant.

### Sequencing analysis.

The sample results presented here are from one plastic section (Fig. 2, flush A.1) and one cast iron pipe section (Fig. 2, flush D.2) and include all the repeated flushing interventions. These samples were used to study in detail the bacterial dynamics over time and differences in bacterial communities between pipe materials.

### Taxonomic analysis.

**FIG 4:**
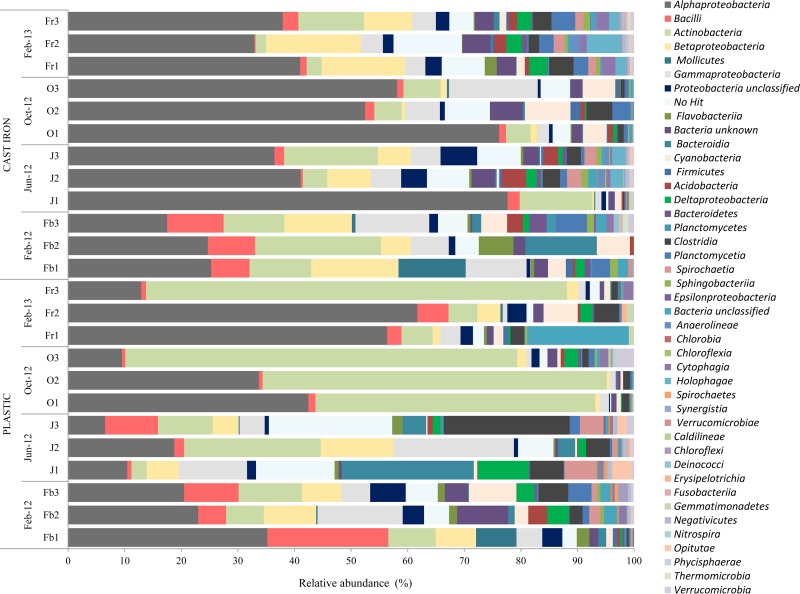
Comparison of the relative abundances of the major phylotypes found in the water collected from the network during flushing, showing differences over time and between pipe materials.

At the class level (Fig. 4), the relative abundance of bacteria changed over time, and differences were observed between pipe materials within the same flushing event. Proteobacteria predominated in all the samples, independently of the pipe material. There was generally more consistency between replicates for the cast iron pipe samples than the plastic pipe samples. Alphaproteobacteria within the Proteobacteria group were highly abundant (based on the average of three biological replicates) in the plastic pipe samples from February 2012 (average, 20%). In June 2012, Clostridia accounted for an average of 22% of the total sequences in the plastic pipe samples, while Actinobacteria were predominant in October 2012 (average, 69%) and February 2013 (average, 74%).

Alphaproteobacteria were prevalent in all the flushing events in cast iron pipe samples, ranging from 17% in February 2012 to 58% in October 2012 (based on the average of three biological replicates). This bacterial group was followed in abundance by Gammaproteobacteria in February 2012 (13%) and October 2012 (15%) and by Actinobacteria in June 2012 (15%) and February 2013 (11%). Other abundant classes in both types of pipe material in all the flushing events were Betaproteobacteria, Bacilli, and Cyanobacteria.

**FIG 5:**
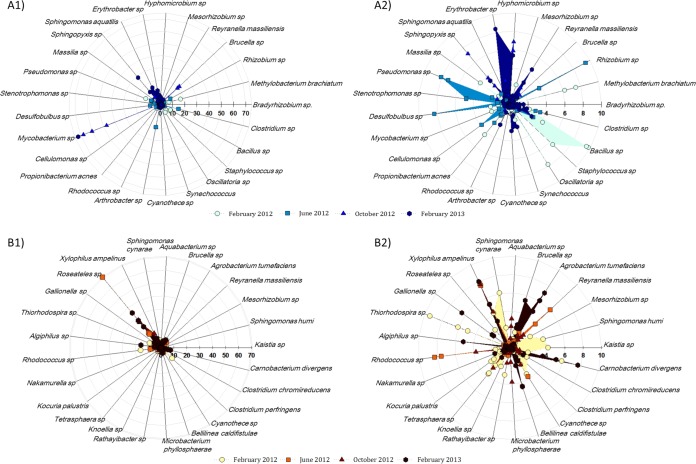
Diagrams showing the relative abundances of the most abundant members of the bacterial communities at a 97% sequence similarity cutoff from samples collected during flushing. (A1) Plastic section showing maximum relative abundance of 75%. (A2) Magnification of image A1 showing maximum relative abundance of 10%. (B1) Cast iron section showing a maximum relative abundance of 70%. (B2) Magnification of image B1 showing a maximum relative abundance of 10%.

Figure 5 shows the most abundant species in both types of pipe material over time. Clear shifts in the dominant species between flushing events and between pipe materials were observed. In the plastic pipe samples in February 2012, Methylobacterium, Bacillus, and Oscillatoria were the dominant genera, while in June, the Pseudomonas, Desulfobulbus, and Rhizobium genera were highly abundant. In October, the dominant species in the samples changed again, and species belonging to Sphingopyxis and Hyphomicrobium dominated the samples, while in February 2013, Erythrobacter and Reyranella spp. dominated the community composition.

In the cast iron pipe samples, Roseateles predominated in all the communities, with the exception of February 2012, when Thiorhodospira was the dominant member of the community. In June, Reyranella and Rhodococcus was highly abundant; in October, members of the Xylophilus genus were highly represented; and in February 2013, samples displayed a high abundance of Agrobacterium, Brucella, and Carnobacterium species.

**FIG 6:**
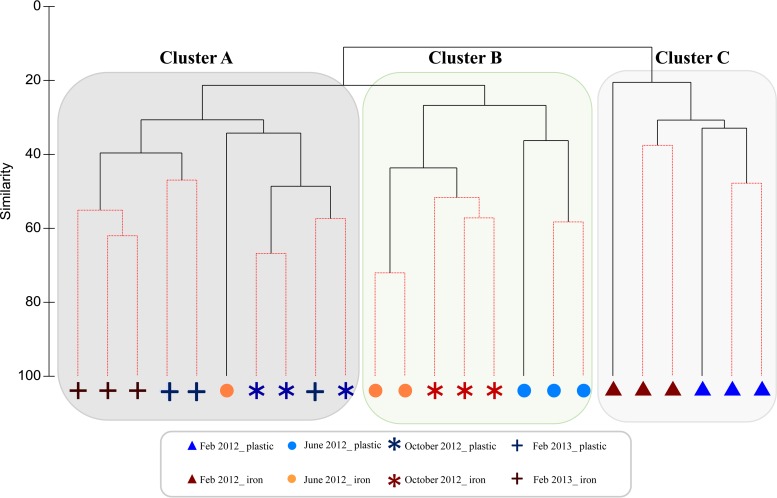
Hierarchical cluster analysis at genus level from samples collected during flushing. SIMPROF, solid (black) lines show clusters significant at a *P* value of <0.05; red lines, non-statistically significant difference.

The hierarchical cluster analysis at the species level (Fig. 6) of the Bray-Curtis matrix, calculated using the relative percentage abundance at a 97% similarity cutoff, confirmed the T-RFLP analysis, with samples clustering by flushing event and according to pipe material. The analysis showed three main statistically significant clusters (SIMPROF, *P* < 0.05). The samples from the first flushing event (February 2012), which occurred after 42 months without any known hydraulic disturbance, formed their own cluster, including two subclusters separated by pipe material (Fig. 6, cluster C). Samples from June 2012 (except one iron sample) and cast iron samples from October 2012 created cluster B; this contained two subclusters, with all the cast iron samples except one (June 2012) and all the plastic ones from June 2012. The third cluster (Fig. 6, cluster A) grouped the plastic samples from October 2012 and samples from both pipe materials from February 2013, along with one cast iron sample from June 2012. Within this cluster, there is further subclustering according to pipe material.

### Diversity and richness indicators.

**FIG 7:**
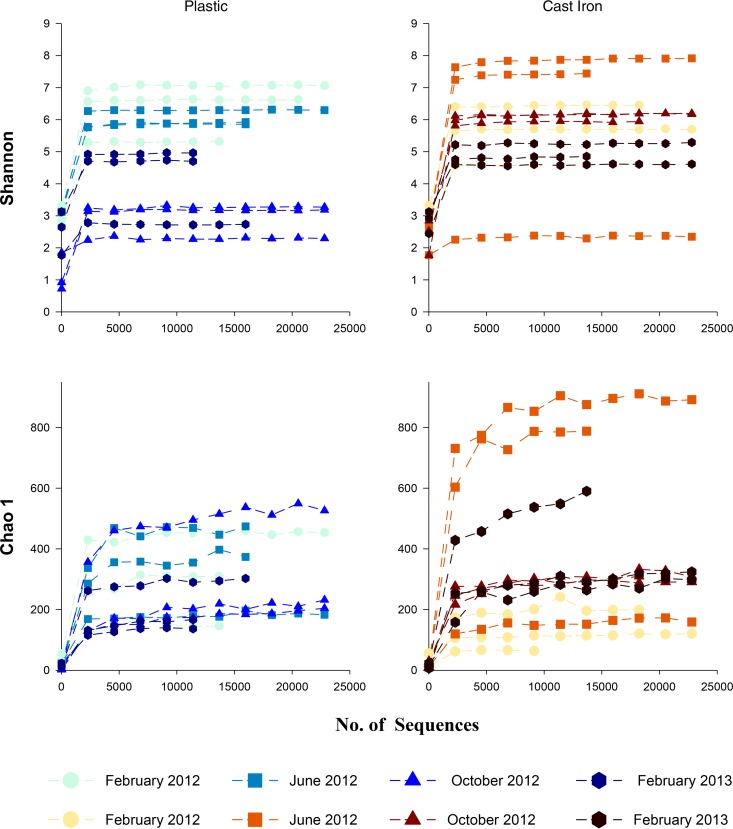
Graphs showing diversity (Shannon index) and richness (Chao1 index) indicators at a 97% sequence similarity cutoff from samples collected during flushing.

In general, no significant differences were detected in diversity and richness indicators between pipe materials; these varied mainly according to flushing event (Fig. 7). The Shannon diversity index values of the plastic pipe samples showed a general reduction in diversity with successive flushes. Diversity was higher in February 2012 and June 2012 and decreased in October 2012 but was mixed in February 2013, generally showing an increase. This is the same order as that observed in the turbidity response (Fig. 2). There was no clear change in diversity for the cast iron pipe samples. The first flushing samples show medium to high levels of microbial diversity, with the highest diversity levels detected for two samples from the second flushing event in June but with one of these repeats having the lowest diversity. Samples from October had less diversity, and those from February 2013 showed the lowest level of diversity (other than the low sample from June 2012). The Chao1 index (richness) for the plastic pipes showed significant variation between replicates, reaching as much as the total range for the initial flushing samples from February 2012. Within this variability, there is a tentative trend for richness to decrease with further flushing events, with the lowest richness in October 2012 (although one sample from this time point had the highest overall richness) and February 2013. For the cast iron samples, richness was more consistent between replicates, with the exception of one sample from June 2012. Richness was initially low in February 2012, with peaks in June 2012 and then decreases showing similar values in October and February 2013.

### Correlations between bulk water physicochemical factors and bacteriological parameters.

Spearman's rank correlations (two-tailed) are shown in Fig. S1 in the supplemental material. Turbidity levels were strongly positively correlated with levels of TOC, metal concentrations (Al, Fe, and Mn), nitrate, and phosphate. Significant positive correlations were detected between turbidity and the relative abundance of several bacterial groups, including Gammaproteobacteria, Bacilli, Mollicutes, Flavobacteriia, and Bacteroidia. The concentration of metals was significantly correlated with levels of TOC, pH, and Cl, and the concentrations of the different metals were also significantly correlated with one another. Metals were also positively correlated with the diversity (Shannon) and the relative abundance of the bacterial groups Gammaproteobacteria, Bacilli, and Flavobacteriia. Phosphate level was positively correlated with turbidity and TOC level and negatively with Cl levels. Phosphate was also the factor that correlated positively with most of the microbiological factors: Shannon index, Chao1 indicator, and the distribution of Proteobacteria, Mollicutes, Cyanobacteria, and Flavobacteriia. Nitrate level was not significantly correlated with diversity and/or richness but was positively correlated with the distribution of Bacilli and Cyanobacteria and negatively correlated with Clostridia, Spirochaetes, and Firmicutes. Sulfate level correlated significantly only with Cyanobacteria and Firmicutes. Free chlorine was negatively correlated with Bacteroidetes and Sphingobacteriia and positively correlated with Clostridia.

## DISCUSSION

### Effect of flushing on turbidity response and biofilm detachment.

Biofilm detachment from pipes due to changes in shear stress has been shown to be particulate (30). It can therefore be theorized that by measuring the turbidity response, the biofilm stability can be inferred, aiding biofilm detachment and rate of accumulation assessment. Turbidity patterns in response to each repeated flushing operation (Fig. 2) were consistent over time for each pipe length across the cast iron and plastic pipes. This suggests continuous and repeatable processes occurring along each pipe length, imparting similar strengths to the accumulated biofilm layers in response to the stepped flushing forces.

Looking at flush A (Fig. 2), in which detailed sequencing analysis was undertaken, the turbidity data in detail show that the plastic pipe section had the highest turbidity levels in February 2012 (anticipated due to the longer accumulation period) and June 2012, with the lowest levels in October 2012 and slightly increased levels again in February 2013 (Fig. 2). The decrease from June to October suggests that increased microbial activity occurs during warmer periods; however, this does not explain the turbidity increase in February 2013. This is possibly due to other seasonal variations, such as organic (or substrate) loading from the source water impacting microbial growth rates. This is supported by the observation in Fig. 7 that the microbial diversity, in particular, follows the same trend with time as the turbidity data for the plastic pipe length and by correlations to nutrient data. However, potential flow events partially mobilizing material between trials can also take place, but with no detailed flow record available at the pipe level, these cannot be confirmed or ruled out.

Similarly, considering flush D (Fig. 2), the cast iron main with detailed sequencing analysis, turbidity data again show an extreme initial turbidity response after 42 months with no known hydraulic disturbance. The subsequent flushing trials, following 4-month accumulation periods, produced very similar turbidity responses peaking at around 10 NTU for each repeat, comparable to the plastic pipe lengths (Fig. 2). This consistency in turbidity response due to repeat flushing of the cast iron pipe length is matched with consistency in microbial diversity and richness (Fig. 7). This supports the hypothesis that corrosion processes dominate the source of material accumulation in cast iron pipes. This is reinforced by higher levels of iron from flushing cast iron pipe (Table 1).

Overall, from this analysis, it can be theorized that microbial behavior in plastic pipes is more influenced by bulk water properties (including temperature and nutrients) than in cast iron sections, where the iron source from localized corrosion has an important influence on the bacteriological composition of mobilized material.

### Dynamics of microbial communities over time and regrowth.

To investigate if bacteria are resilient to episodic flushing events, the bacterial community structure of all the water samples collected during the flushing trial was analyzed by T-RFLPs. Selected samples (plastic versus cast iron) were then used for in-depth sequencing analysis. Similar patterns were observed across both fingerprinting and sequencing, showing that the bacteriological composition of the material attached to the pipes changed over time. This is counter to initial predictions that the microbial communities attached to pipe surfaces were going to recover in a similar manner after flushing, mainly influenced by pipe material and with limited influence by seasonal changes in bulk water. The results, however, demonstrate that microorganisms did not populate the pipe walls in the same way after the flushing, and significant differences were observed in the major members of microbial communities over time. These outcomes may be explained by the influence of two processes: (i) seasonal fluctuations (physicochemical and microbiological) affecting the source water supplied into the distribution system, and (ii) the bacterial community composition of the biofilm remaining attached to the pipe walls after flushing.

### Seasonal fluctuations affecting the source water supplied into the distribution system.

The fingerprinting analysis (Fig. 3) showed differences in the communities between flushing events, including differences between the bulk water inlet samples from the two first flushes. Drinking water treatments plants are not 100% efficient, and an important fraction of the bacteria in the source water is transferred into the distribution system. Consequently, the seasonal changes in factors, such as light, temperature, and nutrients, which affect the bacterial distribution in the source water will be reflected in the bacterial composition of the finished drinking water (31–34). Such seasonal effects will be most apparent for river water sources, such as those studied here, rather than ground water sources or those with large reservoirs, for which changes will be smoothed out by mixing and retention effects.

Differences in community structures between flushing events may have been driven by fluctuations in temperature, as the water in the warmer months was on average 10°C warmer than in the autumn and winter months (Table 1), and it is well known that a rise in temperature can increase the growth rates of heterotrophic bacteria (3). In this study, temperature correlated with the distribution of specific bacterial groups, such as Cyanobacteria (see Fig. S1 in the supplemental material), which were abundant in plastic pipes during the winter months. Similarly, Kormas et al. (35) detected a high abundance of the cyanobacterium Synechococcus in urban DWDS during winter, associated with the occurrence of blooms during autumn, which then sink to the bottom of the reservoir and are not easily detected afterwards. The importance of cyanobacteria in drinking water is related to their capability of producing toxins when high temperatures are reached in surface water reservoirs. What is still open to discussion is why these photosynthetic bacteria are commonly present in biofilms in DWDS. It has been suggested that they might have an alternative nonphototrophic lifestyle (36). Other bacterial groups showing marked temporal variability were Actinobacteria (highly abundant in samples from plastic pipes in June and October) and *Alpha*-, *Beta*-, *Gamma*-, and Deltaproteobacteria.

Several studies have indicated that inorganic nutrients are the limiting factor for microbial growth in DWDS (37–39). In this study, the concentrations of phosphate and metals were the factors that correlated the most with bacteriological parameters (richness and diversity) (see Fig. S1 in the supplemental material). It has been observed that phosphorus availability in drinking water can regulate microbial growth (31) and that, after nonnative bacterial contamination of drinking water, factors like nutrient availability and temperature can influence the survival and die-off patterns of these intruders (40, 41). Phosphate was particularly high in flushing samples from the cast iron pipes, indicating that this nutrient accumulates on the pipe surfaces, is likely trapped, and then is released from biofilms. From these findings, it can be suggested that the control or removal of phosphorus at the treatment stage might restrict microbial growth within DWDS. However, with orthophosphate dosing being a common practice in the United Kingdom to control plumbosolvency and thus protect public health and ensure compliance with increasingly stringent lead regulations, there are competing pressures and risks associated with water treatment that need to be fully understood and considered. Morton et al. (42), using small-scale laboratory experiments, suggested that under certain circumstances, corroded iron pipes can serve as a source of all micronutrients needed for microbial growth, including carbon, nitrogen, and phosphorus. The results reported here, in which a distinctive and more-stable bacterial community was present in cast iron pipe samples, confirm the importance that local corrosion processes have in biofilm composition within DWDS.

Organic carbon is commonly considered to be the limiting factor for microbial growth in DWDS (43). Here, the levels of TOC were positively correlated with some bacterial groups, including Mollicutes and Cyanobacteria. It has been suggested that it is actually assimilable organic carbon (AOC) and not TOC that is the limiting nutrient for microbial growth in DWDS, since this represents the proportion of organic carbon that can be easily assimilable by bacteria and is responsible for heterotrophic bacterial growth (44). AOC was not measured in this study, and further conclusions cannot be drawn concerning the importance of organic carbon as a limiting factor for microbial growth in DWDS.

It is common practice worldwide to maintain a disinfection residual within DWDS to limit bacterial regrowth. The most widely used residual is free chlorine. However, this study confirms the lack of inhibition to biofilm growth, despite adequate levels of residual free chlorine detected in all the samples (Table 1). Chlorine levels correlated negatively only with Bacteroidetes and Sphingobacteriia and correlated positively with Clostridia. The positive correlation between Clostridia and chlorine can be explained by the known ability of this microorganism to form spores that are very resistant to chlorine and other disinfectants. These results confirm that biofilms provide an environment where bacteria are protected from chlorine residuals. An example of the inefficiency of chlorine in controlling biofilms is the high presence of mycobacteria in the plastic pipe samples. Mycobacteria can be resistant to chlorine and are frequently detected in DWDS, and this genus consists of a large number of species that are either nonpathogenic or are opportunistic pathogens (45). Unexpectedly, Mycobacterium spp. were not present (or present in low percentages) in the samples from cast iron, suggesting that plastic materials might promote the growth of these bacteria, or that iron-rich environments inhibit their growth. Kelly et al. (34) also showed that ductile iron pipes in a DWDS disinfected with monochloramine had reduced Mycobacterium abundances within the biofilms. Despite the potential health risk associated with certain species of Mycobacterium, these microorganisms are not included in standard monitoring strategies.

### Effect of bacterial community composition of the biofilm remaining attached on the pipe walls after flushing.

The second main process that might influence the population of microorganisms on the pipe walls is the bacterial composition of the biofilm remaining attached after flushing. Despite the initial hypothesis that the pipes would be colonized in a similar fashion after flushing, it was noticed that different dominant bacterial groups were detached during each flushing trial, particularly for the plastic pipes.

The fraction of material/biofilm remaining attached to the pipe walls will depend on hydrodynamic factors. In this study, pipes were subjected to similar hydraulic regimes over time, and despite the amount of material detached having a tendency to be similar after successive flushes, confirming that hydraulics govern the amount of material mobilized into the water (7, 17), the microbial composition changed markedly, particularly for the plastic pipes. This result suggests that the material remaining attached had a different bacteriological composition each time and/or that different community members were preferentially removed at different times during the study, that is, flushing exerts a selective pressure on the biofilm. However, not only selection but also genetic drift caused by a random detachment of bacteria from the pipe wall can contribute to the reduction in diversity and shift of dominant bacteria.

Flushing possibly selects bacteria that are able to produce greater extracellular polymeric substance (EPS) or with more attachment sites through particular polysaccharides that favor the cell-cell or cell-surface adherence. Previous research on freshwater biofilms by Karwautz and Lueders (46) suggests that the transport of cells into biofilms is primarily influenced by hydrodynamics and resident biofilm topography, but attachment processes are influenced by the biological properties of the planktonic community and by the way in which microorganisms interact with each other. Hence, each time the pipes were flushed, different layers of the biofilm were exposed, and new cell-surface or cell-cell interactions needed to be established to create a new biofilm. The nonparametric correlations reported here show that some bacterial groups were positively correlated and others were not, suggesting that different combinations of bacteria are more likely than others to occur and confirming the existence of specific bacterial interactions. From Fig. 4 and 5, it can be seen that some bacteria are permanent at low percentages and others are transient through the time series of flushing operations. The strong fluctuation between different sampling times reflects the high heterogeneity of communities in each pipe section, particularly the plastic pipe. The level of microbial heterogeneity within biofilms is important, since it has been shown that, for example, pathogen resistance to chlorination is affected by the community biodiversity and interspecies relationships (47). However, further sampling campaigns are needed to corroborate the trend and patterns determined here.

Overall, it can be seen that the action of repeated flushing exposes and removes different layers of the biofilm over time, imposing a selective pressure on the microbial communities and likely favoring the members with greater shear strength properties to remain and start new biofilms. It is observed that flushing reduced bacterial diversity, but several flushes were needed to obtain a clear decrease in the material accumulated on the plastic pipe walls. It was observed that the communities from October and February 2013 were more similar to each other than other samples obtained at different times, suggesting that flushing at regular intervals will in the long term select certain members of the community, favoring a stabilizing effect. Over a period, this could produce biofilms that help avoid mobilization issues, thereby reducing the risk of discoloration and associated opportunistic pathogen release, etc. This suggests that in addition to removing discoloration material, regular repeat flushing could be an effective measure to improve the stability properties of the biofilm and hence decrease risk and/or increase the period required between repeated flushing interventions.

### Pipe material (cast iron and plastic).

Two main trends have been observed in how pipe material affected the mobilization of accumulated material and the development of new biofilm: (i) the two materials, in general terms, had similar levels of diversity and richness but supported different bacterial communities; and (ii) seasonality in plastic pipe samples was more marked than in cast iron pipe samples, suggesting that the colonization of plastic pipes is influenced more by the seasonal dynamics occurring in the source water. Samples from the plastic pipes clearly showed shifts in diversity over time, but in cast iron, these shifts are less marked, and despite changes in the dominant species, a less-heterogeneous community was observed at the genus level.

It could be suggested that biofilm layers are easier to remove from the relatively smooth surface of plastic pipes, leaving more available uncolonized surfaces for bacteria from the bulk water to attach. This might explain why the microbial community composition of plastic pipes is markedly affected by seasonality. On the other hand, the less-marked seasonal effect on cast iron pipe samples might be due to the influence of the nutrient source provided by the corroding iron on community composition. Further, it is possible that interaction with the specialized bacterial community protected within the microenvironment of corrosion scales, which promotes iron bacteria (48), promotes a more-permanent community. This specialist community would not be exposed to disinfection residual and hence would remain viable to influence biofilm growth. Conversely, anaerobes and/or microorganisms sensitive to chlorine previously protected within biofilm EPS would be exposed, leaving the new community to be more influenced by the bulk water, as observed in the plastic pipes. In accordance with this, it has been shown that cast iron pipes favor the presence of bacteria able to metabolize iron and manganese compounds, thus supporting a community of metabolically specialized microorganisms and potentially restricting the interactions with new microbial members (8).

Henne et al. (6), who studied mature biofilms in DWDS, suggested that the primary bacterial colonization depends on surface material, but then the coexistence of the communities over the years will influence the composition by the exchange of bacteria and the influence of adjacent biofilm communities. After the first flushing event in which mature biofilm was removed, distinct bacterial communities were observed between the two types of pipe material despite the pipes being connected in the same DMA. Similarly, Chao et al. (49), who studied drinking water biofilms grown on annular reactors, showed considerable differences between biofilms formed on stainless steel and plastic. There is controversy in the literature regarding how pipe material can affect microbial communities, with different studies debating if plastic or metals support more or less microbial diversity or growth (50–52). This study shows a clear effect of material on microbial community structure. To summarize, both materials are attractive for biofilm growth but for different types of bacteria, with plastic being more influenced by effects of seasonality from the incoming water.

### Conclusion.

This research has described bacterial community dynamics in the material mobilized from pipes after a series of successive seasonal flushing events. Bacterial communities were shown not to repopulate in the same way and were highly variable, fluctuating with season and pipe material. These results suggest that the bacterial dynamics over time were influenced by (i) differences in the supplied water (including temperature, nutrients, etc.) and (ii) the material left attached on the pipe wall following flushing that favors the presence or absence of certain bacterial groups. Both plastic and cast iron pipe materials are shown to be similarly susceptible to bacterial colonization but by different bacteria. Compared to plastic pipes, cast iron pipes showed a less-diverse community and less-marked seasonal variation in association with a greater and more-repeatable turbidity response. Conversely, plastic pipes supported a more-diverse community influenced by seasonal changes in the bulk water.

Turbidity, metals, and phosphate were the main factors correlated with the distribution of bacteria in the samples. With the lack of inhibition of biofilm development due to chlorine residual in the pipes, this suggests that limiting inorganic nutrients other than organic carbon at the treatment level might be a viable component in strategies to manage biofilms. The research showed that repeat flushing exerts beneficial selective pressure; therefore, it is a viable long-term advantageous management option.

Overall, this work highlights the complexity and persistence of microbe-induced material accumulation within DWDS. New monitoring approaches should be implemented that incorporate other nonstandardized physicochemical factors and microorganisms to have a better representation of the real conditions in DWDS. Similarly, monitoring strategies should consider incorporating a higher-throughput screening of the general pipe conditions on a seasonal basis to detect the presence of persistent and/or seasonal microorganisms.

## Supplementary Material

Supplemental material
