# Supplementary material for: Dynamics of Biofilm Regrowth in Drinking Water Distribution Systems
Source: Appl Environ Microbiol. 2016 Jun 30;82(14):4155–68. doi: 10.1128/AEM.00109-16 (PMC4959196; doi:10.1128/AEM.00109-16)
Supplement: Supplemental material [file supp_82_14_4155__index.html]

Supplemental material 

# Dynamics of Biofilm Regrowth in Drinking Water Distribution Systems

## Supplemental material

- Supplemental file 1 -

  Spearman rank correlations between physicochemical and environmental variables from samples collected during flushing (Fig. S1).

  PDF, 77K
