# Supplementary material for: Dynamics of Biofilm Regrowth in Drinking Water Distribution Systems
Source: Appl Environ Microbiol. 2016 Jun 30;82(14):4155–68. doi: 10.1128/AEM.00109-16 (PMC4959196; doi:10.1128/AEM.00109-16)
Supplement: Supplemental material [file AEM.00109-16_zam999117256so1.pdf]

Figure S1: Spearman rank correlations between physico-chemical and environmental variables from samples collected during flushing. Vales marked with an asterisk (*) and highlighted are showing significant correlations.

	Chao1	Shannon	Turbidty	TOC	Temp	pH	Cl	Al	Fe	Mn	Nitrate	Phosphate	Sulphate	Alphaproteobacteria	Betaproteobacteria	Gammaproteobacteria	Deltaproteobacteria	Bacilli	Actinobacteria	Mollicutes	Flavobacteriia	Bacteroidia	Cyanobacteria	Acidobacteria	Bacteroidete	Planctomycetes	Clostridia	Planctomycetia	Spirochaetia	Sphingobacteriia	Actinobacteria	Anaerolineae	Cytophagia	Holophagae	Spirochaetes	Chloroflexi	Firmicutes	Gemmatimonadetes
Chao1	1.000																																					
Shannon	.186	1.000																																				
Turbidty	-.118	.391	1.000																																			
TOC	-.034	.336	.479*	1.000																																		
Temp	-.003	.273	-.216	.167	1.000																																	
pH	.140	-.378	-.373	-.395	-.719**	1.000																																
Cl	-.189	-.208	-.192	-.286	.310	.108	1.000																															
Al	-.181	.418*	.515*	.786**	.381	-.778**	-.452*	1.000																														
Fe	.320	.586**	.743**	.714**	.024	-.443*	-.524**	.690**	1.000																													
Mn	.152	.541**	.635**	.524**	.190	-.707**	-.667**	.786**	.857**	1.000																												
Nitrate	-.123	-.139	.491*	.381	-.786**	.287	-.476*	.214	.333	.167	1.000																											
Phosphate	.473*	.444*	.611**	.667**	-.119	-.192	-.429*	.524**	.905**	.619**	.452*	1.000																										
Sulphate	-.147	-.234	.287	-.167	-.952**	.719**	-.119	-.381	-.071	-.286	.738**	.071	1.000																									
Alphaproteobacteria	.254	-.115	-.379	-.095	-.168	.437*	-.229	-.334	-.050	-.129	-.102	-.037	.060	1.000																								
Betaproteobacteria	.209	.387	.423*	-.084	-.244	-.104	-.242	.173	.320	.302	.323	.423*	.265	-.407*	1.000																							
Gammaproteobacteri	.235	.664**	.640**	.389	.218	-.498*	-.110	.470*	.704**	.620**	.011	.609**	-.187	-.261	.473*	1.000																						
Deltaproteobacteria	.097	.409*	.163	-.194	-.081	.032	.100	-.013	0.000	-.050	.053	.102	.202	-.365	.690**	.197	1.000																					
Bacilli	-.324	.205	.703**	.439*	-.205	-.301	-.355	.533**	.565**	.544**	.499*	.462*	.236	-.100	.200	.270	-.03	1.000																				
Actinobacteria	-.301	-.451*	-.141	.179	.152	-.221	.005	.236	-.194	-.045	.095	-.218	-.189	-.329	-.277	-.217	-.446*	.132	1.000																			
Mollicutes	.034	.277	.519**	.450*	-.026	-.229	-.180	.352	.541**	.441*	.203	.421*	.031	.147	.072	.301	-.114	.543**	-.157	1.000																		
Flavobacteriia	-.154	.446*	.643**	.281	-.037	-.368	-.205	.489*	.542**	.497*	.308	.534**	.108	-.357	.593**	.520**	.413*	.534**	-.278	.094	1.000																	
Bacteroidia	-.048	.478*	.534**	.100	.229	-.443*	.060	.313	.423*	.423*	-.121	.315	-.126	-.450*	.347	.594**	.253	.308	-.190	.004	.576**	1.000																
Cyanobacteria	.077	.084	.484*	.462*	-.478*	.233	-.252	.102	.483*	.218	.549**	.475*	.452*	.320	-.156	.157	-.087	.472*	-.160	.375	.097	-.070	1.000															
Acidobacteria	.501*	.573**	.061	.072	.049	-.085	-.330	.145	.390	.331	-.013	.464*	-.125	-.047	.476*	.291	.384	.067	-.370	.178	.293	.407*	-.109	1.000														
Bacteroidete	.326	.404	.120	.103	-.214	.011	-.422*	.182	.264	.254	.314	.336	.139	-.202	.604**	.089	.562**	.026	-.260	.099	.177	.027	-.031	.524**	1.000													
Planctomycetes	.151	.399	.173	.198	-.008	-.089	-.113	.208	.266	.169	.166	.361	.008	-.353	.443*	.134	.286	.087	-.175	.191	.364	.059	-.148	.385	.582**	1.000												
Clostridia	-.259	.307	-.122	-.439*	.213	.025	.418*	-.260	-.328	-.273	-.455*	-.370	-.045	-.127	.085	.018	.538**	-.100	-.337	-.327	.087	.276	-.158	.027	-.058	-.105	1.000											
Planctomycetia	.465*	.443*	.119	.074	-.184	.188	-.081	-.050	.265	.042	.176	.444*	.171	-.180	.468*	.230	.437*	.097	-.200	.138	.088	.037	.162	.547**	.527**	.590**	.185	1.000										
Spirochaetia	.090	.551**	-.075	-.246	.384	-.186	.187	.035	-.027	.005	-.451*	-.005	-.250	-.245	.445*	.272	.668**	-.180	-.402	-.223	.405*	.446*	-.486*	.445*	.251	.313	.583**	.190	1.000									
Sphingobacteriia	.395	.367	.045	.079	-.263	.087	-.481*	.147	.255	.223	.352	.384	.166	-.030	.643**	.285	.450*	.040	-.132	-.033	.309	-.089	-.007	.423*	.661**	.480*	-.190	.523**	.226	1.000								
Actinobacteria	-.080	.411*	.057	.186	.268	-.264	-.268	.351	.236	.333	-.185	.066	-.227	-.124	-.124	.093	.034	.159	.036	.006	.017	.171	.022	.219	-.061	.078	.357	.264	.209	-.112	1.000							
Anaerolineae	.550**	.258	-.445*	-.209	.011	.289	-.249	-.180	-.026	-.092	-.178	.146	-.069	.173	.263	-.082	.419*	-.365	-.301	-.371	-.065	-.002	-.197	.628**	.427*	.125	.265	.489*	.443*	.421*	.321	1.000						
Cytophagia	.286	.074	-.173	-.356	-.124	.169	-.079	-.172	-.164	-.124	-.008	-.040	.132	-.372	.428*	-.081	.562**	-.272	-.010	-.354	.020	.170	-.229	.401	.375	.232	.199	.416*	.401	.390	.163	.537**	1.000					
Holophagae	.410*	.120	-.389	-.598**	.082	.153	.059	-.390	-.285	-.191	-.390	-.194	-.106	-.100	.443*	-.011	.434*	-.585**	-.209	-.532**	-.034	.175	-.581**	.354	.370	.127	.275	.165	.574**	.345	-.122	.611**	.612**	1.000				
Spirochaetes	-.005	.182	.219	-.117	.062	-.066	.218	.055	.045	-.031	-.055	.117	.107	-.294	.424*	.331	.659**	.093	-.110	-.251	.360	.351	.073	-.060	.023	-.095	.540**	.205	.480*	.203	.089	.176	.462*	.271	1.000			
Chloroflexi	.180	.109	-.406*	-.192	-.110	.291	-.169	-.107	-.245	-.245	.054	-.067	.107	-.195	.326	-.197	.496*	-.339	.072	-.590**	-.012	-.112	-.270	.299	.454*	.303	.235	.416*	.443*	.528**	.267	.730**	.664**	.559**	.289	1.000		
Firmicutes	-.025	.480*	.085	.017	.583**	-.559**	.177	.348	.077	.221	-.417*	-.017	-.478*	-.448*	.217	.299	.472*	.129	.080	-.135	.264	.515*	-.288	.273	.120	.157	.470*	.215	.623**	.156	.368	.155	.387	.221	.547**	.270	1.000	
Gemmatimonadetes	-.207	-.366	-.420*	-.181	.060	.204	.218	-.315	-.507*	-.384	-.264	-.649**	-.061	.398	-.682**	-.499*	-.265	-.388	.053	-.035	-.625**	-.439*	.069	-.450*	-.356	-.516**	-.038	-.540**	-.320	-.370	-.099	-.227	-.158	-.176	-.254	-.194	-.157	1.000
Verrucomicrobia	.193	.155	-.166	-.301	.263	-.151	.157	-.053	-.146	-.061	-.303	-.069	-.231	-.330	.246	-.010	.510*	-.080	-.116	-.079	.047	.189	-.234	.370	.234	.264	.396	.430*	.441*	.145	.163	.355	.642**	.263	.373	.325	.571**	-.147
